# Novel Characteristics of *Trypanosoma brucei* Guanosine 5'-monophosphate Reductase Distinct from Host Animals

**DOI:** 10.1371/journal.pntd.0004339

**Published:** 2016-01-05

**Authors:** Tomoaki Bessho, Tetsuya Okada, Chihiro Kimura, Takahiro Shinohara, Ai Tomiyama, Akira Imamura, Mitsuru Kuwamura, Kazuhiko Nishimura, Ko Fujimori, Satoshi Shuto, Osamu Ishibashi, Bruno Kilunga Kubata, Takashi Inui

**Affiliations:** 1 Laboratory of Biological Macromolecules, Graduate School of Life and Environmental Sciences, Osaka Prefecture University, Sakai, Osaka, Japan; 2 Laboratory of Veterinary Pathology, Graduate School of Life and Environmental Sciences, Osaka Prefecture University, Izumisano, Osaka, Japan; 3 Laboratory of Toxicology, Graduate School of Life and Environmental Sciences, Osaka Prefecture University, Izumisano, Osaka, Japan; 4 Laboratory of Biodefense and Regulation, Osaka University of Pharmaceutical Sciences, Takatsuki, Osaka, Japan; 5 Laboratory of Organic Chemistry for Drug Development, Faculty of Pharmaceutical Sciences, Hokkaido University, Sapporo, Japan; 6 AU/NEPAD Agency Regional Office for Eastern and Central Africa, Nairobi, Kenya; Liverpool School of Tropical Medicine, UNITED KINGDOM

## Abstract

The metabolic pathway of purine nucleotides in parasitic protozoa is a potent drug target for treatment of parasitemia. Guanosine 5’-monophosphate reductase (GMPR), which catalyzes the deamination of guanosine 5’-monophosphate (GMP) to inosine 5’-monophosphate (IMP), plays an important role in the interconversion of purine nucleotides to maintain the intracellular balance of their concentration. However, only a few studies on protozoan GMPR have been reported at present. Herein, we identified the GMPR in *Trypanosoma brucei*, a causative protozoan parasite of African trypanosomiasis, and found that the GMPR proteins were consistently localized to glycosomes in *T*. *brucei* bloodstream forms. We characterized its recombinant protein to investigate the enzymatic differences between GMPRs of *T*. *brucei* and its host animals. *T*. *brucei* GMPR was distinct in having an insertion of a tandem repeat of the cystathionine β-synthase (CBS) domain, which was absent in mammalian and bacterial GMPRs. The recombinant protein of *T*. *brucei* GMPR catalyzed the conversion of GMP to IMP in the presence of NADPH, and showed apparent affinities for both GMP and NADPH different from those of its mammalian counterparts. Interestingly, the addition of monovalent cations such as K^+^ and NH_4_^+^ to the enzymatic reaction increased the GMPR activity of *T*. *brucei*, whereas none of the mammalian GMPR’s was affected by these cations. The monophosphate form of the purine nucleoside analog ribavirin inhibited *T*. *brucei* GMPR activity, though mammalian GMPRs showed no or only a little inhibition by it. These results suggest that the mechanism of the GMPR reaction in *T*. *brucei* is distinct from that in the host organisms. Finally, we demonstrated the inhibitory effect of ribavirin on the proliferation of trypanosomes in a dose-dependent manner, suggesting the availability of ribavirin to develop a new therapeutic agent against African trypanosomiasis.

## Author Summary

Only a limited number of therapeutics for human African trypanosomiasis also known as African sleeping sickness is available today, and it narrows the choice of the drugs to escape from the side effects and the emergence of drug-resistant pathogens. The parasitic protozoa *Trypanosoma brucei* is the causative reagent of African trypanosomiasis, and is infective to various mammalian species. *T*. *brucei* and its mammalian hosts share almost the same metabolic machinery, and therefore it is important to understand the differences in biochemical properties of the metabolic enzymes between *T*. *brucei* and its hosts. Here we report that guanosine 5’-monophosphate reductase (GMPR) of *T*. *brucei* showed apparent differences in its primary structure and biochemical properties from those of its host counterparts, and was more sensitive to purine nucleotide analogs such as monophosphate forms of ribavirin and mizoribine than were the host GMPRs. Furthermore, ribavirin prevented the proliferation of trypanosomes *in vitro*. Our present findings may imply the availability of ribavirin and/or its derivatives in a treatment of African trypanosomiasis.

## Introduction

In general, purine nucleotides are synthesized *de novo* from their precursors such as amino acids and ribose 5-phosphate, and are also produced from purine bases and ribose 5-phosphate through a salvage pathway. Guanosine 5’-monophosphate reductase (GMPR) catalyzes the reductive deamination of guanosine 5’-monophosphate (GMP) to inosine 5’-monophosphate (IMP) in the presence of NADPH, a route to recycle guanine nucleotides into adenine nucleotides [1]. GMPR has been identified in various species from bacteria to mammals including parasitic protozoa [2], and has been structurally characterized by X-ray crystallography, which indicated that GMPR belongs to the family of (β/α)_8_ barrel proteins also known as TIM barrel proteins. It is also known that GMPR shows high similarities in amino acid sequence and structure to inosine 5’-monophosphate dehydrogenase (IMPDH), the enzyme catalyzing the NAD^+^-dependent oxidation of IMP to xanthosine 5’-monophosphate (XMP); nevertheless, GMPR and IMPDH are generally distinguished by the cystathionine β-synthase (CBS) domain, which is well conserved in IMPDHs but absent in GMPRs [1]. Recent studies have demonstrated that the catalytic mechanism of *E*. *coli* GMPR follows an ordered bi-bi kinetic mechanism [3], and that the GMPR reaction uses the same intermediate E-XMP* as IMPDH, but in this reaction the intermediate reacts with ammonia instead of water [4]. However, detailed studies on GMPRs have been performed only on human and bacterial enzymes, and so the GMPRs in other organisms including protozoa are still poorly defined.

*Trypanosoma brucei* is a protozoan parasite and the causative agent of African trypanosomiasis, a vector-borne parasitic zoonosis known as African sleeping sickness in humans and as nagana disease in cattle. Nearly all the protozoa are incapable of *de novo* purine biosynthesis and depend on the purine salvage pathway, which has been regarded as an attractive chemotherapeutic target of parasitemia [5]. Indeed, *T*. *brucei* lacks the enzymatic machinery for the *de novo* synthesis of purine nucleotides, and therefore it solely depends on salvaging purines acquired from the extracellular environment for survival [6]. Recently, several groups have investigated the genomic information of *T*. *brucei*, and a gene registered as Tb927.5.2080 was annotated as a putative *T*. *brucei* GMPR (TbGMPR) in TriTrypDB and GeneDB [7,8]; however, the molecular identification and characterization of TbGMPR still remain to be made.

In this study, we examined the GMPR activity of the recombinant protein of the Tb927.5.2080 gene, and identified the subcellular localization of TbGMPR in *T*. *brucei* bloodstream forms. Furthermore, we compared the characteristics of TbGMPR with those of GMPRs of host animals in terms of their enzymatic kinetics and structures and found that ribavirin 5'-monophosphate, a purine nucleotide analog, was a inhibitor of *T*. *brucei* but not of its host GMPRs.

## Materials and Methods

### Materials

All chemicals were purchased from Wako Pure Chemical Industries, Ltd. (Osaka, Japan) or Nacalai Tesque Inc. (Kyoto, Japan), except for GMP (Sigma-Aldrich Japan K.K., Tokyo, Japan) and RMP (Toronto Research Chemicals, Ontario, Canada). MZP was synthesized as described previously [9].

### Trypanosomes

*T*. *brucei brucei* ILTat 1.4, a strain with a monomorphic bloodstream form *in vitro*, was cultured in HMI-11 medium [10], a modified IMDM medium (Sigma-Aldrich Japan), which was supplemented with the following (mM): hypoxanthine (1.0), thymidine (0.16), cysteine (1.5), pyruvate (1.0), bathocuproine sulfonate (0.05), 2-mercaptoethanol (0.2), and 10% fetal bovine serum. Cultures were maintained in a humidified atmosphere of 5% CO_2_ and 95% air at 37°C. A pleomorphic GUTat 3.1 strain was cultured and maintained as described above.

### RT-PCR

Total RNA from *T*. *brucei* was prepared with Sepasol-RNA I Super (Nacalai Tesque). The cDNA was synthesized from the total RNA by using ReverTraAce and oligo-d(T)_20_ primers (TOYOBO, Osaka, Japan). The sequences of the primers were as follow: TbGMPR primers, 5’-GTTCCTATCGTTGGGCAAAA-3’ and 5’-ATACAAGGTACACCCCTGCG-3” α-tubulin primers, 5’-TCAAGTGCGGTATCAACTAC-3’ and 5’-AGTGCTGCAAGGTCTTCAC-3’. PCR reactions were performed with a thermal cycler PC-300 (ASTEC Co., Ltd., Fukuoka, Japan) operated under the following conditions: 95°C for 3 min; 30 cycles of 95°C for 10 s, 55°C for 10 s, and 72°C for 30 s, with an additional extension at 72°C for 5 min. The reaction products were analyzed by electrophoresis on agarose gels, and subsequently by PCR-direct sequencing.

### Expression and purification of recombinant TbGMPR

The coding region of the TbGMPR gene was amplified by using the genomic DNA of *T*. *brucei* as a template, with primers 5’-CGGAATTCATGTCCTTCAATGAATCGGCA-3’ and 5’-CGGTCGACTTAAAGTTTGGCAACACCGTGA-3’. The PCR product was cloned between *Eco* RI and *Sal* I sites of the expression vector pGEX 6P-1 (GE Healthcare Japan Co., Tokyo). The recombinant TbGMPR was expressed as an N-terminal glutathione *S*-transferase fusion protein in *E*. *coli* BL21 (DE3). The water-soluble fraction of the cell extract was applied onto an Affi-Gel Blue Gel 100–200 mesh (Bio-Rad) equilibrated with 50 mM Tris-HCl (pH 8.0) containing 150 mM NaCl, 1 mM EDTA and 1 mM DTT. The column was washed with the same buffer containing 1 M KCl, and the recombinant protein was eluted with the buffer containing 3 M KCl. The eluted protein was further purified with glutathione-Sepharose 4B and PreScission protease (GE Healthcare Japan) according to manufacturer’s instructions. The purified protein was dialyzed against 50 mM Sodium phosphate buffer (pH 7.0) containing 100 mM KCl, 3 mM EDTA, 1 mM DTT, and 5% glycerol. The concentration of the purified recombinant protein was determined with BCA Protein Assay Reagent (Thermo Fisher Scientific) by using bovine serum albumin as a standard, and the purity was analyzed by SDS-PAGE.

### HPLC analysis of GMPR reaction mixture

The mixture for the GMPR reaction consisted of 50 mM Tris-HCl (pH 8.0), 100 mM KCl, 3 mM EDTA, 500 μM GMP, and 500 μM NADPH. Following pre-incubation at 37°C, the reaction was initiated by adding recombinant enzyme at 0.5 ng/ml. The reaction was terminated by filtering out the enzyme from the reaction mixture with VIVASPIN 500 (Sartorius AG, Goettingen, Germany), and 10 μl of the filtrate was subsequently subjected to analysis with an HPLC system (LaChrom Elite L2000, Hitachi High-Technologies Co., Tokyo, Japan) equipped with a Cadenza CD-C18 column (2.0 mm x 150 mm x 3 μm, Imtakt Co., Kyoto, Japan). The following mobile phases were prepared separately: solvent A contained PIC-A reagent (Waters) diluted according to the manufacturer's manual. Solvent B consisted of acetonitrile containing 0.1% trifluoroacetic acid. Both solvents were pumped at a flow rate of 190 μl/min under the following gradient protocol: The concentration of solvent B was gradually increased from 2.5 to 20% for the first 15 min, from 20 to 50% for the next 6 min, and finally kept at 70% for 10 min. The eluents were monitored through a UV detector (Waters) with a wavelength at 254 nm. Commercial IMP, GMP, NADPH, and NADP^+^ were used as standards to identify and quantify the corresponding compounds in the reaction mixture.

### Preparation and evaluation of anti-TbGMPR polyclonal antibody

Immunization of a rabbit with the purified recombinant TbGMPR as an antigen, and preparation of the antiserum were performed by Medical and Biological Laboratories Co., Ltd. (Nagoya, Japan). Whole IgG was fractionated from the antiserum by ammonium sulfate precipitation followed by Ab-Rapid SPiN EX column chromatography (ProteNova Co. Ltd., Kagawa, Japan). Crude lysates of *T*. *brucei* were prepared in RIPA buffer and subsequent centrifugation. The lysates were separated on a 10% SDS–PAGE gel and transferred to a PVDF membrane (Bio-Rad). The membrane was then incubated overnight at 4°C with 5 μg/ml of the anti-TbGMPR polyclonal IgG in Tris-buffered saline with 0.05% Tween-20 (TBST) containing 5% skim milk (Wako). After having been washed with TBST, the blots were incubated with anti-rabbit IgG conjugated with horseradish peroxidase (Cell Signaling Technologies, Danvers, MA). The immunoreactivities were visualized by using ImmunoStar Zeta (Wako) and LAS4000 imaging device (GE Healthcare Japan).

### Immunostaining of TbGMPR in *T*. *brucei* cells

*T*. *brucei* (5.0 x 10^5^ cells) were washed with PBS and fixed with 5% formalin in PBS for 15 min on ice, and then adhered to PLATINUM-coated glass slides (Matsunami Glass Ind., Ltd., Osaka, Japan). Cells were permeabilized for 20 min with 0.3% (v/v) Triton X-100 in PBS, followed by blocking for 10 min with 50% (v/v) fetal bovine serum in PBS. They were then incubated with polyclonal rabbit anti-TbGMPR (5 μg/ml) for 45 min and, after a serial washing with PBS, were incubated for 45 min with secondary antibody conjugated with Alexa Fluor 488 (goat anti-rabbit IgG; Life Technologies) at a 1: 200 dilution. Subsequently, the cells were washed with PBS and mounted in glycerol containing DAPI (1 μg/ml). As a negative control, staining was performed as described above but in the absence of primary antibody. Differential interference contrast and fluorescence optical images were captured under nonsaturating conditions by using a confocal laser scanning microscope LSM-700 (Zeiss).

For immunoelectron microscopy, *T*. *brucei* (1.3 x 10^8^ cells) were fixed with 4% paraformaldehyde and 0.1% glutaraldehyde in 0.1 M phosphate buffer (PB, pH 7.6) at 4°C for 30 min. They were then washed with 0.1 M PB and embedded in 1% agarose. Next, the samples were sequentially treated with 20% sucrose in PB for cryoprotection, embedded in TISSU MOUNT compound (Shiraimatsu co., LTD., Osaka, Japan), and sectioned at a 10-μm thickness on a cryostat. After having been dried on glass slides, the sections were rinsed in PBS containing 0.1% Triton X-100 and treated with 10% normal goat serum in PBS for 10 min. Then they were incubated with anti-TbGMPR antibody (10 μg/ml) at 4°C overnight. After washing in PBS, the sections were incubated at 4°C overnight with nanogold-conjugated anti-rabbit IgG (Nanoprobes, NY) at a 1: 200 dilution. After a washing with PB, the gold particles were enhanced by using an HQ Silver enhancement kit (Nanoprobes) and then post fixed with 1% osmium tetraoxide in 0.1M PB at 4°C for 1 h. After the samples had been dehydrated by passage through a graded series of ethanol, they were embedded in epoxy resin. Ultrathin sections were cut and examined by electron microscopy (Hitachi High-Technologies Co.).

### Determination of kinetic parameters of GMPRs

GMPR activity was measured at 35°C in a buffer containing 50 mM sodium phosphate buffer (pH 7.0) including 100 mM KCl, 3 mM EDTA, and 1 mM DTT. The concentrations of GMP (0 to 1,000 μM) or NADPH (0 to 150 μM) were varied to determine the kinetic parameters for each compound. The activities were measured as NADPH consumption as described above. The initial velocity data (n = 3) were fitted to the Michaelis-Menten equation by using Origin 6.0 (Microcal, Northampton, MA) or IGOR Pro 6.3 (WaveMetrics, Inc., OR) [11].

### Temperature and pH dependency of TbGMPR activity

The activity of recombinant TbGMPR was examined at different temperatures in 50 mM sodium phosphate buffer (pH 7.0), with the ionic strengths adjusted to 0.2 by adding NaCl. Reactions were initiated by adding 100 nM enzyme to each buffer containing 1 mM GMP and 200 μM NADPH. Measurements of TbGMPR activity at different pHs were performed in 50 mM sodium phosphate buffer (pH 6.5 to 8.0) or 50 mM sodium borate buffer (pH 8.0 to 9.0). GMPR activity was defined as NADPH consumption, which was monitored at 340 nm (*ɛ*_340_ = 6220 M^-1^·cm^-1^) with a DU-800 spectrophotometer (Beckman Coulter). All measurements were performed in triplicate.

### Activation of GMPR by monovalent cations

The effects of monovalent cations on the activity of recombinant TbGMPR were examined at 35°C in 50 mM sodium phosphate buffer (pH 7.0) containing 1 mM GMP, 200 μM NADPH, and 150 mM monovalent chloride (NaCl, KCl or NH_4_Cl). The reaction was initiated by the addition of 100 nM enzyme, and the NADPH concentrations in the reaction mixture were monitored as described above. The GMPR activities of human and bovine enzymes in the presence of 150 mM NaCl or KCl were examined by using the same procedure, except that the NADPH concentration was 800 μM.

### Inhibitor assays of GMPRs

Inhibitory activities of RMP and MZP toward GMPRs were tested by the same procedure applied for the determination of kinetic parameters, except that the concentrations of GMP (1,000 μM) and NADPH (200 μM for TbGMPR, and 800 μM for mammalian GMPRs) were fixed unless otherwise specified. Various concentrations of RMP or MZP were added 5 min prior to the reaction initiation, and subsequent NADPH consumption was monitored with a spectrophotometer. The initial velocity data (n = 3) at the various concentrations of the compounds were fitted to the Hill equation to calculate the IC_50_ values. For RMP, the data were plotted on Lineweaver-Burk plots, and fitted to a competitive inhibition equation to calculate the *K*_i_ values [11].

### Examination of anti-trypanosomal activity of ribavirin *in vitro*

*T*. *brucei* GUTat 3.1 cultured in HMI-11 medium was inoculated to the fresh medium and placed on 96-well plates at a density of 2 x 10^3^ cells/mL in the presence of ribavirin. PBS was used as a control. The final concentrations of the drug were ranged from 1 to 1,000 μM. The parasites were incubated for 72 h in 5% CO_2_ and 95% air at 37°C. Four hours prior to finish the incubation, 1/10 vol. of 1.25 mg/mL resazurin was added. The number of the parasites in each well after treatment was measured by colorimetric method on a plate reader Benchmark Plus (BIO-RAD), by subtracting the absorbance at 600 nm from those at 570 nm. The cell densities of the drug treatment groups were obtained relative to the control group (n = 4).

### Statistical analysis

In the study on the GMPR activities in the presence of the monovalent cations, the significance of differences in the enzymatic activities between the NaCl group and others was analyzed by using one-way ANOVA followed by Dunnett’s test. The differences were considered as significant when the *p* values were below 0.05 (n = 3).

## Results

### Molecular characterization of the Tb927.5.2080 gene in *T*. *brucei* cells

The molecular characterization of GMPR in trypanosomatids had remained unreported until now. Our sequence alignment analysis showed that all the putative GMPRs of trypanosomatids, including the Tb927.5.2080 gene of *T*. *brucei*, shared a unique insertion of about 130 amino acid residues; and a domain search on PROSITE [12] revealed that this insertion possibly corresponded to a tandem repeat of CBS domains (S1 and S2 Figs), which are absent in human, bovine, and *E*. *coli* GMPRs.

**Fig 1:**
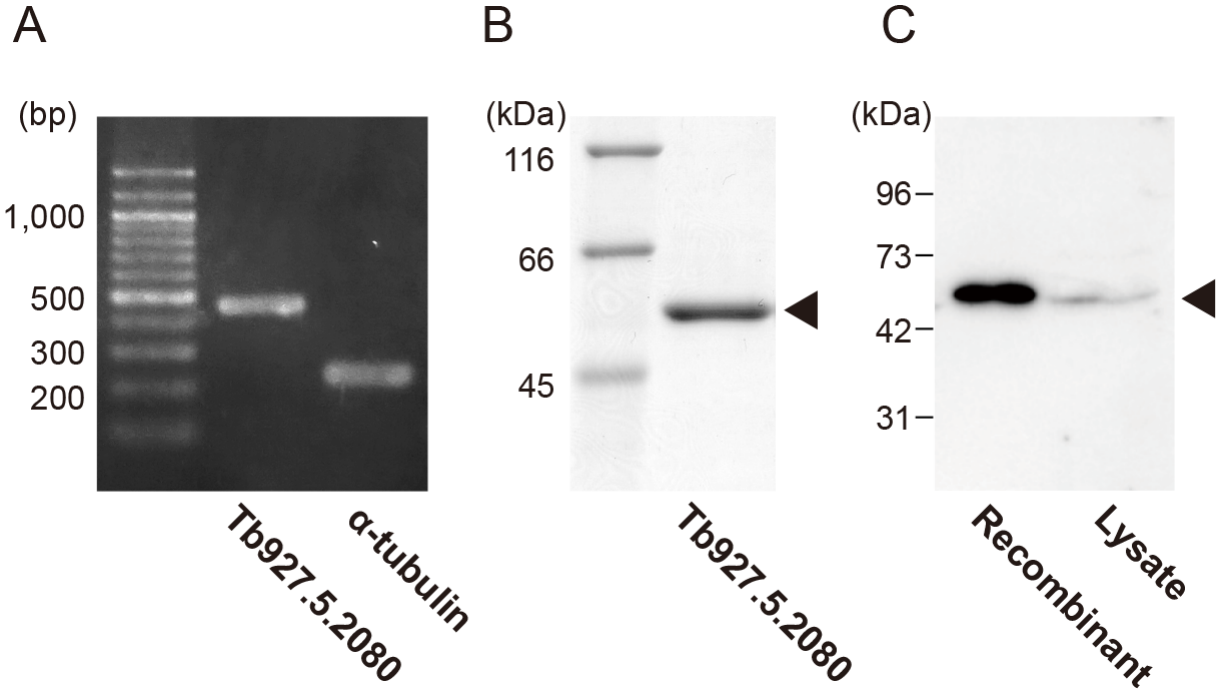
Detection of Tb927.5.2080 gene product from *T*. *brucei* cells. A. A coding sequence region within Tb927.5.2080 gene was amplified by RT-PCR with *T*. *brucei* total RNA as a template. The expression of α-tubulin was also examined as a positive control. B. SDS-PAGE was performed with 5 μg of the purified recombinant protein. A single band with molecular weight of approximately 53 kDa (arrowhead) was observed after CBB staining. C. Whole cell lysate of *T*. *brucei* (20 μg protein) and recombinant protein (50 ng) were subjected to Western blot analysis with the polyclonal antibody prepared as described in Materials and Methods. A single band was detected at a position of approximately 53 kDa (arrowhead) from each sample.

We performed RT-PCR analysis to clarify endogenous mRNA expression of the Tb927.5.2080 gene in *T*. *brucei*. Total RNA was extracted from bloodstream forms of *T*. *brucei* in culture, and used as a template. RT-PCR analysis with primers specific for the Tb927.5.2080 gene produced a single-sized product (Fig 1A), and subsequent sequencing analysis of this PCR product revealed that it was certainly derived from the mRNAs of the Tb927.5.2080 gene.

We then generated a recombinant TbGMPR by use of an *E*. *coli* expression system and found that the purified recombinant protein exhibited a single band on an SDS-PAGE gel with the expected molecular weight of approximately 53 kDa (Fig 1B). The purified protein was used as an antigen to raise a polyclonal antibody against TbGMPR. By Western blotting against the whole cell extracts of cultured *T*. *brucei* with prepared antibody, a single band was detected at a position of approximately 53 kDa which corresponded to the size of the recombinant TbGMPR (Fig 1C).

### Examination of GMPR activity of the Tb927.5.2080 gene product

**Fig 2:**
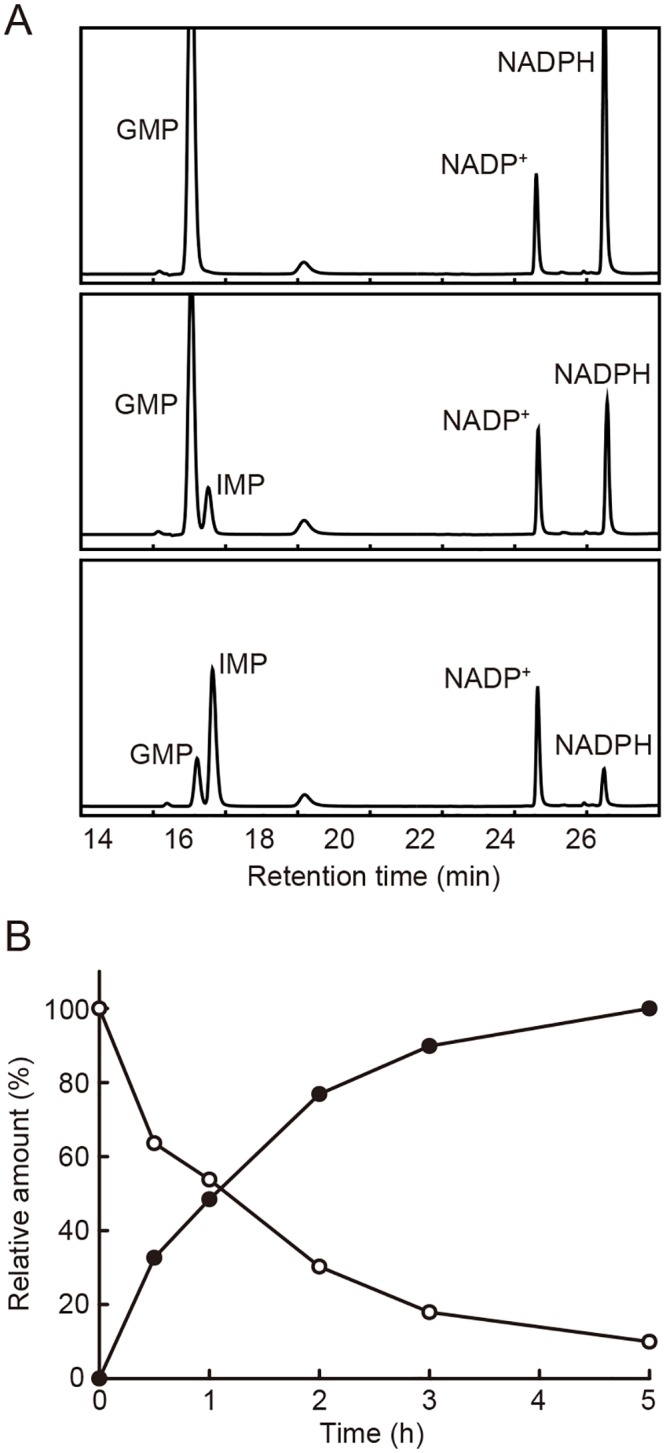
HPLC analysis of GMPR activity of the Tb927.5.2080 gene product. A. HPLC chromatograms of the products of the reaction with the recombinant protein encoded by the Tb927.5.2080 gene. The mixtures were sampled at 0 h (top), 1 h (middle), and 5 h (bottom) after initiating the reaction. Note that the peak heights of GMP and NADPH became lower while those of IMP and NADP^+^ became higher as the reaction proceeded. B. Consumption of GMP and formation of IMP during the reaction were quantified based on the HPLC data. The amounts of GMP (open circles) and IMP (closed circles) were expressed in percent relative to their highest values.

To examine the GMPR activity of the Tb927.5.2080 gene product, we incubated the recombinant protein with GMP and NADPH, a substrate and a co-enzyme of GMPRs, and subsequently analyzed the reaction mixture by HPLC at various time points. The absorbance at retention times of 15.0 and 27.2 min (corresponding to those of authentic GMP and NADPH, respectively) decreased in a time-dependent manner; and conversely, the absorbance at 15.6 and 24.4 min (corresponding to those of authentic IMP and NADP^+^, respectively) increased with the same time course (Fig 2). These results indicate that the recombinant protein of Tb927.5.2080 gene catalyzed the conversion of GMP into IMP in the presence of NADPH, meaning that Tb927.5.2080 actually encoded the GMPR of *T*. *brucei*. Based on the above results, we designated the Tb927.5.2080 gene and its encoded protein as TbGMPR.

### Subcellular localization of GMPR protein in *T*. *brucei* bloodstream forms

**Fig 3:**
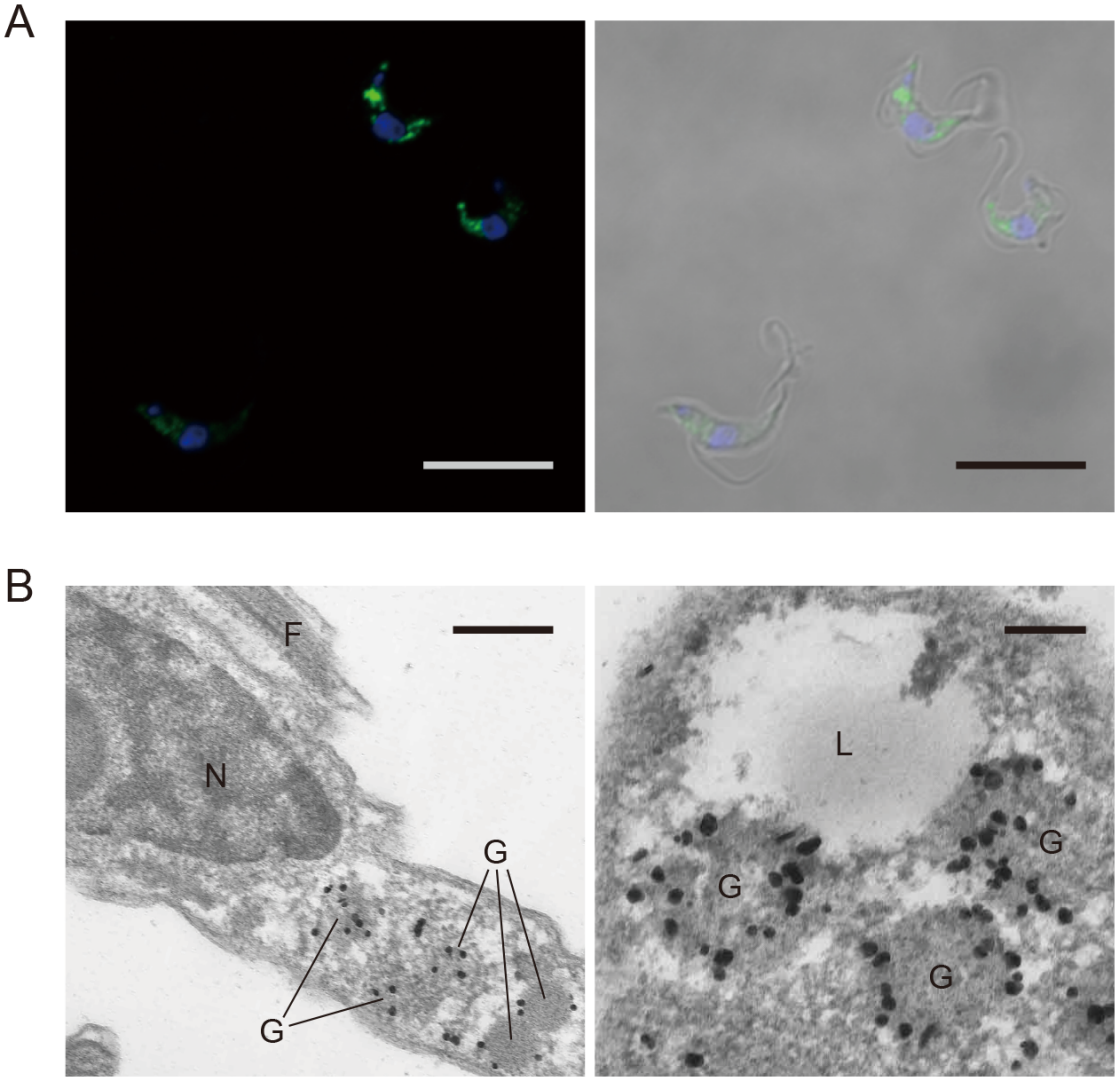
Localization of TbGMPR in bloodstream forms of *T*. *brucei*. Subcellular localization of TbGMPR in *T*. *brucei* was examined by immunostaining with the polyclonal anti-TbGMPR antibody. A. Green fluorescence of TbGMPR immunoreactivities was observed as granular forms throughout the cells (left panel). The nuclei were stained with DAPI (blue). The right panel shows the differential interference contrast image merged with the left image. Scale bars: 10 μm. B. TbGMPR immunoreactivities were visualized with silver-enhanced particles under transmission electron microscopy, and they were mainly found in the glycosomes. G, glycosome; N, nucleus; L, lysosome; F, flagellum. Scale bars: 0.5 (left) and 0.2 μm (right).

Immunofluorescent staining with the anti-TbGMPR polyclonal antibody showed that the fluorescent signals were distributed throughout the cells of *T*. *brucei* bloodstream forms in culture and seemed to be located in vesicle-like organelles (Fig 3A). Further anatomical study was performed by immunoelectron microscopy, and the majority of the immunoreactivities were found in glycosomes (Fig 3B). All GMPRs in trypanosomatids possess a peroxisomal-targeting signal (Ala/Ser-Lys-Leu) on their C-terminus (S2 Fig), which is commonly found in glycosomal proteins of *T*. *brucei* [13,14]. Taken together, all our results clearly demonstrate that GMPR was consistent to be localized in the glycosomes of *T*. *brucei* bloodstream forms, though we did not use antibodies against *bona fide* glycosomal markers.

### Steady-state kinetics of GMPRs of *T*. *brucei* and mammals

**Fig 4:**
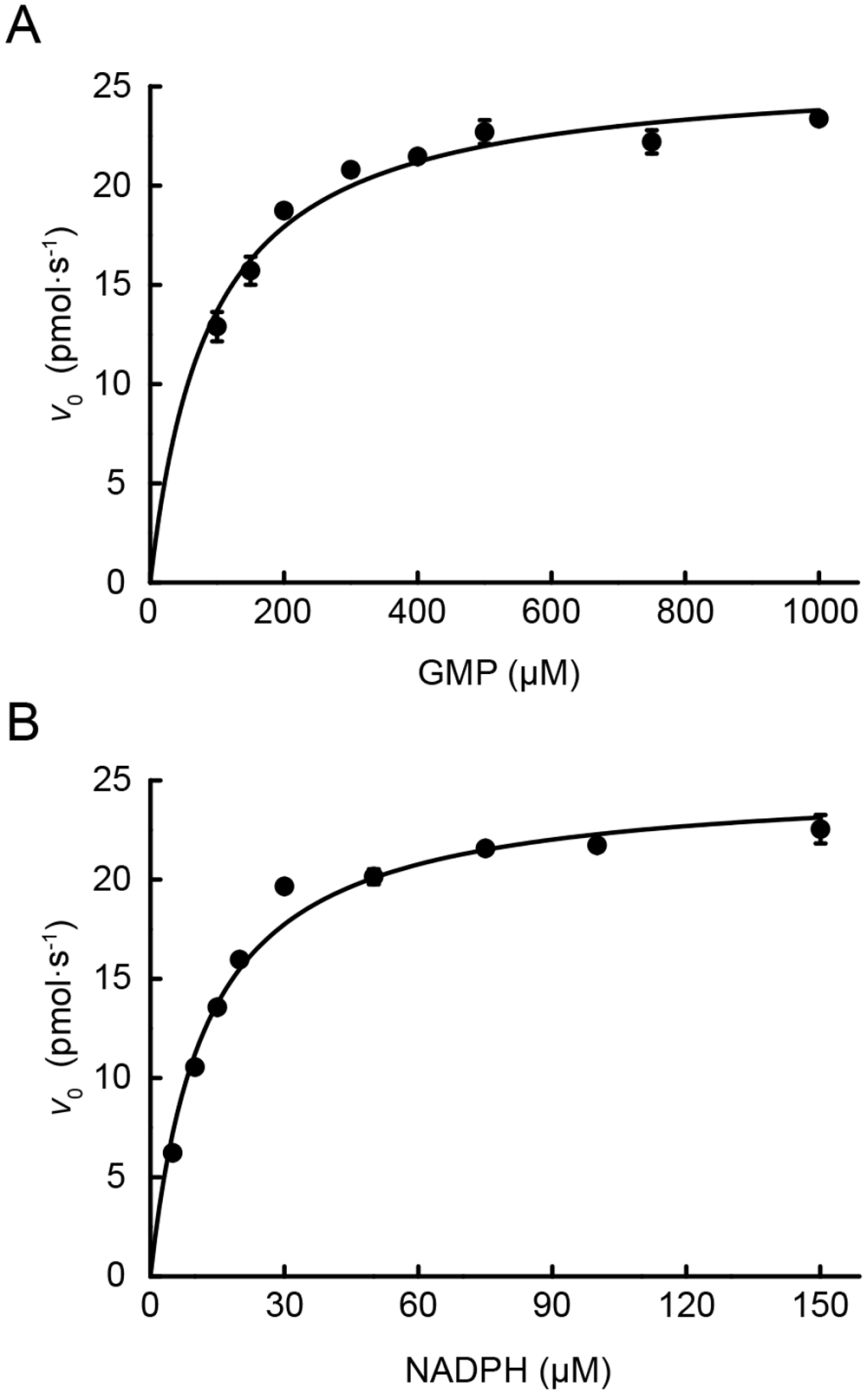
Steady-state kinetics of TbGMPR. A. Plot of the initial velocity *versus* the GMP concentration in the presence of 200 μM NADPH. B. Plot of initial velocity *versus* NADPH concentration in the presence of 1 mM GMP. The data were fitted to the Michaelis-Menten equation. The values are expressed as the mean ± S.D.

In order to characterize the kinetic properties of TbGMPR, we calculated kinetic constants for the recombinant enzyme. The initial velocities at a various concentrations of GMP or NADPH were collected and processed according to Michaelis-Menten equation (Fig 4). The steady-state kinetic parameters of TbGMPR were determined to be the following: *k*_cat_ value, 0.519 ± 0.012 s^-1^, and *K*_m_ values for GMP and NADPH, 89.3 ± 9.0 μM and 12.3 ± 0.8 μM, respectively (Table 1). We also determined the steady-state kinetic parameters of recombinant GMPRs of human and bovine enzymes, as summarized in Table 1. Based on these data, TbGMPR had lower affinity for GMP than either human or bovine GMPRs. On the other hand, NADPH bound to TbGMPR with higher affinity than did the mammalian GMPRs. Consequently, the differences in the affinities for the substrate and co-enzyme of GMPRs among species raised the *k*_cat_ value of TbGMPR about 2-fold in comparison to mammalian values.

**Table 1:** Parameters of steady-state kinetics of GMPRs.

	*k*_cat_ (s^-1^)	*K*_m GMP_ (μM)	*K*_m NADPH_ (μM)
TbGMPR	0.519 ± 0.012	89.3 ± 9.0	12.3 ± 0.8
HsGMPR1	0.284 ± 0.006	22.1 ± 2.2	34.8 ± 4.3
HsGMPR2	0.265 ± 0.016	17.8 ± 3.5	29.3 ± 3.2
BtGMPR1	0.243 ± 0.004	13.5 ± 1.3	54.1 ± 5.0
BtGMPR2	0.296 ± 0.009	22.6 ± 4.2	62.1 ± 7.0

### Temperature and pH dependency of TbGMPR activity

**Fig 5:**
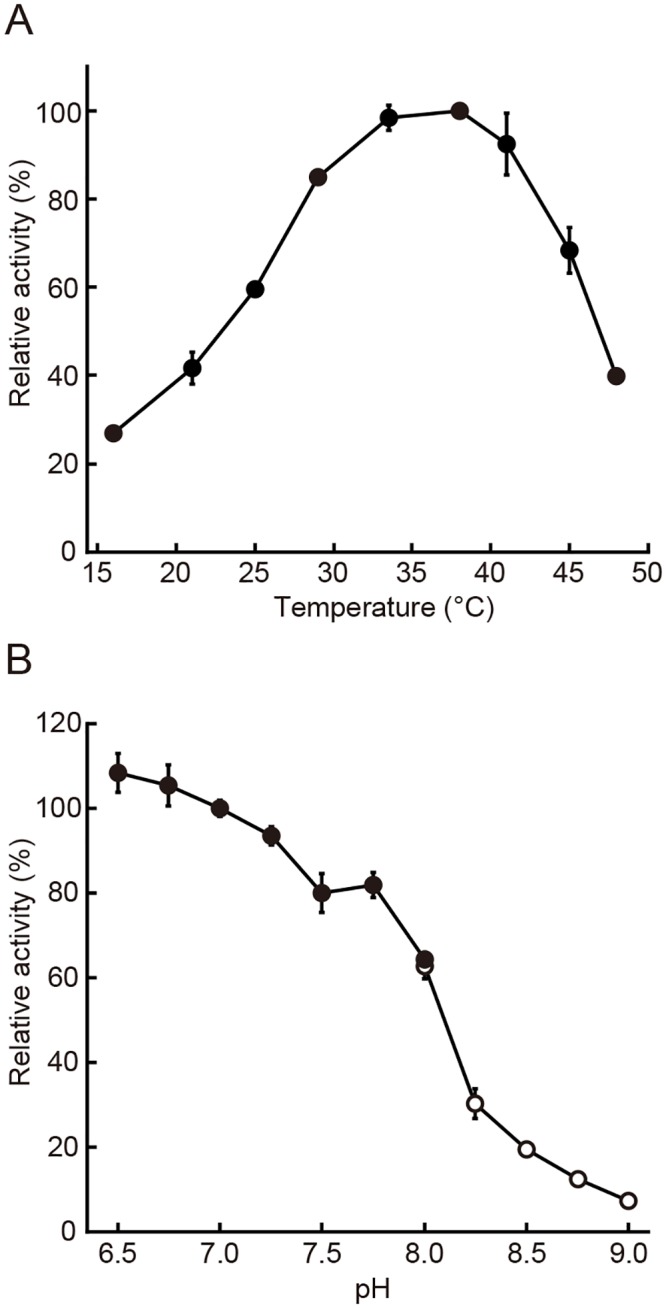
TbGMPR activities at different temperatures or pH conditions. A. Relative GMPR activity is plotted against temperature. The plots were normalized by setting the value at 38°C to 100%. B. Relative GMPR activity is plotted against pH. The activity at pH 7.0 was set to 100%. The activity at different pHs were measured in 50 mM sodium phosphate buffer (pH 6.5 to 8.0; closed circles) or 50 mM sodium borate buffer (pH 8.0 to 9.0; open circles) as described in Materials and Methods. The activity at pHs lower than 6.5 was not examined because of instability of NADPH. The values are expressed as the mean ± S.D.

The enzymatic properties of the recombinant TbGMPR at various temperatures and pHs were assessed to determine the optimal reaction conditions for analysis of steady-state kinetics of the protein. In terms of temperature dependency, TbGMPR exhibited the maximum enzymatic activity of 26.5 ± 0.8 (nM·s^-1^) at 38°C (Fig 5A). The enzymatic activity of the recombinant TbGMPR increased as the pH was reduced from pH 9.0 down to 6.5 (Fig 5B). The isoelectric point of TbGMPR was expected to be pI 9.7 from its amino acid sequence, and this value fell within those of common glycosomal proteins ranging from pI 8.8 to 10.2 [15]. It has been reported that the glycosomal pH of *T*. *brucei* procyclic forms is around 7.4 [16]. Our results with the previous findings suggest that TbGMPR is able to exhibit its enzymatic activity in *T*. *brucei* glycosomes.

### Monovalent cation dependency of GMPR activity

**Fig 6:**
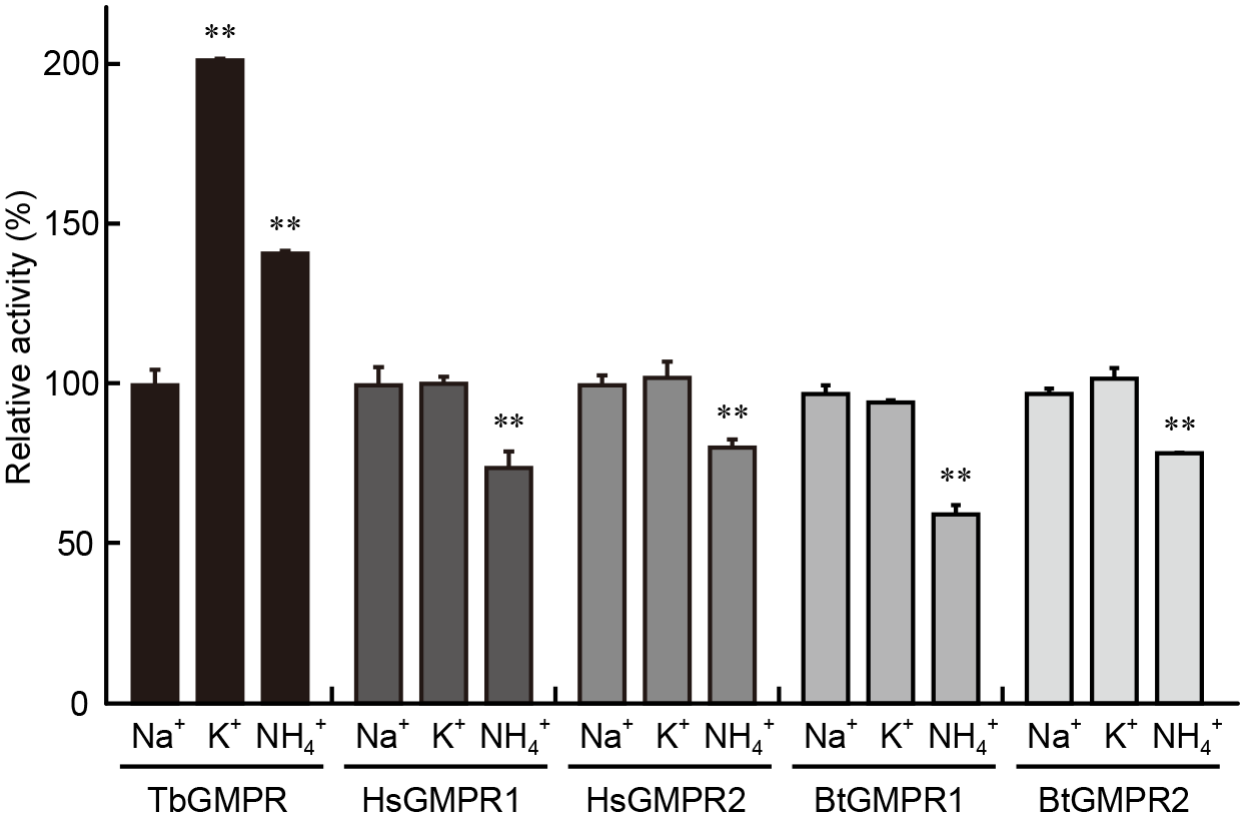
GMPR activities in the presence of monovalent cations. The GMPR activities of *T*. *brucei*, human, and bovine enzymes were measured in the presence of the chloride form of each monovalent cation. The activities were normalized by the value of NaCl group for each enzyme. The values are expressed as the mean ± S.D. **; *p* < 0.01, NaCl group vs. others.

**Fig 7:**
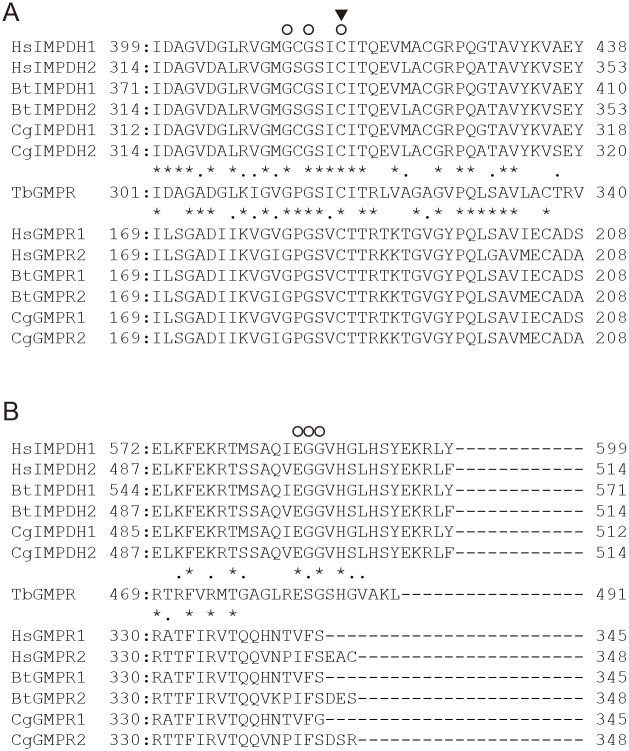
Amino acid sequences of putative K^+^-binding sites of TbGMPR. The amino acid sequence of TbGMPR was aligned against Cys loops (A) and C-terminal regions (B) of mammalian IMPDHs or GMPRs. Circles and a triangle show K^+^-binding residues and a catalytic Cys residue, respectively, described previously for HsIMPDH2 and CgIMPDH2 [17,18]. Asterisks above and below the TbGMPR sequence indicate the amino acid residues identical to mammalian IMPDHs and GMPRs, respectively. Dots indicate the amino acid residues with chemically similar characteristics. Hs, human; Bt, bovine; Cg, Chinese hamster. NCBI accession numbers are as follow: NP_899066 for HsIMPDH1, NP_000875 for HsIMPDH2, NP_001071309 for BtIMPDH1, NP_001029588 for BtIMPDH2, XP_007636735 for CgIMPDH1, XP_007650832 for CgIMPDH2, XP_007621414 for CgGMPR1, and EGW11548 for CgGMPR2.

We examined the effects of various monovalent cation chlorides such as NaCl, KCl, and NH_4_Cl on the activity of the recombinant TbGMPR. The activity of TbGMPR in the presence of NaCl was determined as 21.7 ± 1.2 nM·s^-1^ under the conditions employed here (Fig 6). Interestingly, TbGMPR activities were increased to 43.8 ± 0.2 and 30.6 ± 0.5 nM·s^-1^ when NaCl was replaced with KCl and NH_4_Cl, respectively. Furthermore, neither human nor bovine GMPRs were activated in the presence of KCl instead of NaCl. As opposed to the activity of TbGMPR, the activities of the mammalian GMPRs in NH_4_Cl were significantly decreased, being 61.1 to 81.6% of those in NaCl. TbGMPR showed significantly higher homologies to mammalian IMPDHs than to other GMPRs on BLAST analysis (S1 Table). The sequence alignment of IMPDHs and GMPRs revealed that the amino acid residues that interact with the potassium ion were conserved in TbGMPR and in all IMPDHs but not in other GMPRs examined in this study (Fig 7), supporting our findings of TbGMPR activation by potassium ions. This was the first observation of the activation of GMPR in the presence of monovalent cations.

### Inhibitory effects of purine nucleotide analogs on GMPRs

**Fig 8:**
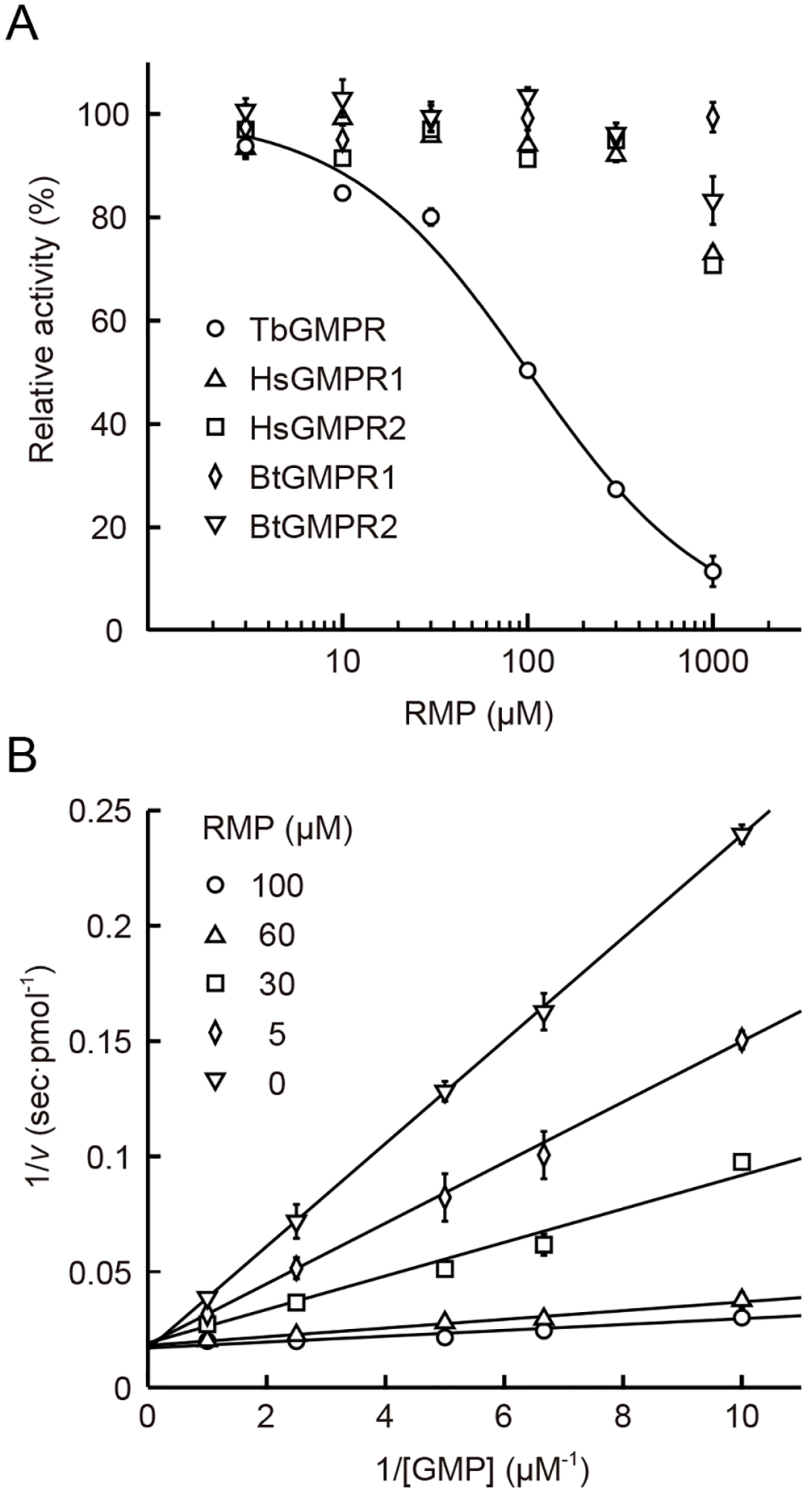
Inhibitory kinetics of RMP for TbGMPR. A. Dose-response curves of RMP-mediated inhibition of GMPRs. The activities of TbGMPR at different concentrations of RMP were fitted to the Hill equation to calculate the IC_50_ value. Note that RMP failed to inhibit recombinant enzymes of both human and bovine orthologs at the concentrations examined. The activity of each GMPR in the absence of RMP was set to 100%. B. Lineweaver-Burk plots for TbGMPR activity in the presence of various concentrations of RMP. The data were fitted to the equation of a competitive inhibition model. The values are expressed as the means ± S.D. from 3 independent experiments.

Next we evaluated the inhibitory effects on GMPRs of ribavirin 5’-monophosphate (RMP) and mizoribine 5’-monophosphate (MZP), which are purine nucleotide analogs known as potent inhibitors of IMPDHs that share a similar conformation with GMPRs [4,11]. Inhibitory kinetic analysis revealed that RMP inhibited TbGMPR with an IC_50_ value of 101.8 ± 0.9 μM; however, no or little inhibition of human and bovine GMPRs was observed in the presence of RMP (Fig 8A). Lineweaver-Burk plots showed that RMP inhibited TbGMPR in a competitive manner, and the *K*_i_ value was determined to be 4.46 ± 0.46 μM (Fig 8B). MZP also inhibited TbGMPR (IC_50_ = 23.7 ± 1.9 μM), though it also exhibited moderate inhibition of HsGMPR1 (119.9 ± 16.1 μM) and HsGMPR2 (69.3 ± 7.8 μM).

### Examination of ribavirin as an anti-trypanosomal drug *in vitro*

**Fig 9:**
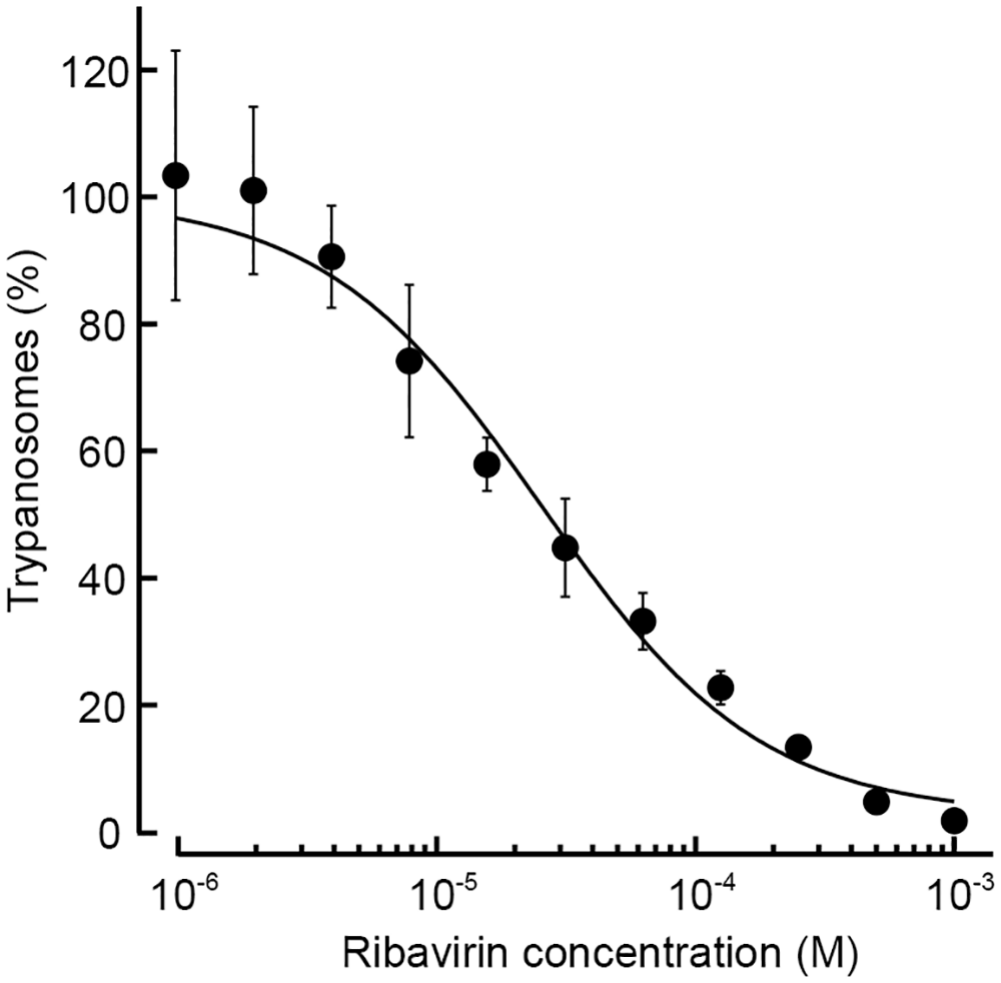
Inhibitory effect of ribavirin on *T*. *brucei* in culture. Relative densities of trypanosomes were plotted against each concentration of ribavirin in the culture medium. The density of trypanosomes with vehicle treatment was set to 100%. Each plot was fitted to the Hill equation to calculate the IC_50_ value. The values are expressed as the means ± S.D. from 4 independent experiments.

To investigate the inhibitory effect of ribavirin on trypanosome proliferation, trypanosomes were cultured with various concentrations of ribavirin for 72 h and the numbers of the living cells relative to the vehicle treatment were examined by using resazurin. The proliferation of trypanosomes was inhibited by the addition of ribavirin in a dose-dependent manner (Fig 9). The curve fitting to Hill equation revealed that the IC_50_ value of ribavirin against trypanosomes was estimated to be 25.4 ± 3.9 μM.

## Discussion

In this study, we identified the GMPR in *T*. *brucei* for the first time and demonstrated its unique properties, which were distinct from those of GMPRs in host animals in terms of protein structure, enzymatic property, and cytological distribution.

So far, 2 genes in *T*. *brucei*, Tb10.61.0150 and Tb927.5.2080, have been annotated as IMPDH in the NCBI database, whereas none have been identified as GMPR. Previously we showed that the former gene encodes IMPDH of *T*. *brucei* [11]; however, the product of the latter gene has not been characterized at the molecular level. Nonetheless, some databases such as TritrypDB and GeneDB annotate this gene as a putative GMPR inferred from its sequence orthology [7,8]. We observed the mRNA expression of Tb927.5.2080 gene in cultured *T*. *brucei*, and this was well corresponding to the previous findings obtained from transcriptome analyses [19–23]. Our present study clearly demonstrated the GMPR activity of the recombinant protein of the Tb927.5.2080 gene, and so this is the first molecular identification of GMPR in *T*. *brucei*. On the other hand, TbGMPR showed no enzymatic activity to catalyze IMP to XMP, despite TbGMPR had higher homology to IMPDHs than to GMPRs of mammals on our BLAST search (S1 Table). All IMPDHs annotated to date share 5 amino acid residues for IMP-binding [18], and yet Tyr411 and Gly415 (HsIMPDH2 numbering) of those residues are substituted in TbGMPR to Ile398 and Ala402, respectively. These differences may account for the lack of IMPDH activity in TbGMPR.

We also examined the expression and localization of TbGMPR and found that TbGMPR was dominantly localized in the glycosomes of *T*. *brucei* bloodstream forms in culture. Glycosomes are peroxisome-like vesicles that contain the machineries for glycolysis and some parts of purine nucleotide metabolism in trypanosomatids. TbGMPR possesses a signal peptide sequence to allow translocation into glycosomes. TbGMPR has been found in the glycosomal extract prepared from the procyclic forms of *T*. *brucei* [24]; however, recent proteome analysis failed to detect the enzyme in the glycosomes of bloodstream forms [25]. Therefore, our present data are the first observation that TbGMPR is consistently localized in the glycosomes of *T*. *brucei* bloodstream forms.

In this study, we showed that TbGMPR possessed a unique insertion of a single pair of putative CBS domains that were absent in GMPRs of species besides trypanosomatids. The CBS domain pairs have been found in proteins such as cystathionine β-synthase, AMP-activated protein kinase, and IMPDH; and they are known to bind adenine nucleotides to function as sensors for intracellular metabolites [26]. AMP-activated protein kinase is involved in switching ATP generation and consumption by sensing AMP concentrations through its CBS domains, and mutations in these domains have been shown to cause severe heart defects known as Wolff-Parkinson-White syndrome [27,28]. CBS domains of IMPDHs of various species including *T*. *brucei* [29] are able to bind adenine nucleotides [28,30], and a mutation in the CBS domain is associated with retinitis pigmentosa in humans [31,32]. These previous findings together with our results suggest that the CBS pair of GMPR, as that of IMPDH, functions as a sensor of adenosine nucleotides in *T*. *brucei*, which could be a novel function specific to trypanosomal GMPRs.

We showed here that TbGMPR was activated by the addition of monovalent cations such as K^+^ and NH_4_^+^. No GMPRs so far except IMPDHs have been found to be activated in the presence of K^+^ and NH_4_^+^, though *E*. *coli* GMPR was reported to catalyze the opposite reaction in the presence of NH_4_^+^ to form GMP from IMP [4]. Our study of the recombinant GMPR enzyme of *T*. *brucei* is the first to show the enhancement of GMPR activity in the presence of monovalent cations. Both GMPR and IMPDH in mammals are known as homotetramers; and especially in IMPDH, the Cys loop existing between the β6 strand and the α6 helix forms a half of the monovalent cation-binding site, whereas the another half is contributed by the end of a C-terminal α-helix from an adjacent monomer [1,18]. Our sequence alignment of the ion-binding sites of mammalian IMPDHs with the corresponding regions of GMPRs showed that the TbGMPR had higher similarity to IMPDHs than to mammalian GMPRs. This observation is consistent with our findings showing that TbGMPR, but neither human nor bovine GMPRs, was activated by monovalent cations, suggesting that either K^+^ or NH_4_^+^ functions as a molecular “lubricant” to facilitate a conformational change in TbGMPR, as suggested in the case of IMPDHs of various species [18]. These structural and functional similarities between GMPRs and IMPDHs in trypanosomatids might have occurred during the evolution from their ancestors, and further investigation might provide a new knowledge regarding the molecular phylogenetics of protozoa.

Our studies with purine nucleotide analogs and recombinant GMPRs clearly showed that RMP was an competitive inhibitor of TbGMPR, with a *K*_i_ value similar to that observed for the inhibition of *T*. *brucei* IMPDH (*K*_i_ = 3.2 ± 0.16 μM) [11]. It has been found that GMPR of *E*. *coli* is inhibited by RMP with a *K*_i_ value of 98 ± 25 μM [4], though this value is about 20-fold higher than that for TbGMPR (4.46 ± 0.46 μM). Interestingly, RMP showed no or little inhibition of human and bovine GMPRs, whereas MZP, another purine nucleotide analog tested, inhibited the activities of both *T*. *brucei* and human GMPRs. These compounds are known to inhibit IMPDHs in a competitive manner by interfering with the IMP binding site [18]; and therefore, it is of considerable interest that these compounds also share the same binding site on TbGMPR. Our findings that RMP preferentially inhibited TbGMPR rather than mammalian GMPRs provide the knowledge to aid in the design of novel species-specific inhibitors of GMPRs. In addition, TbGMPR had higher *K*_m_ and *k*_cat_ values for GMP as compared with human and bovine GMPRs or with *E*. *coli* GMPR [4]. These findings suggest that the reaction center of trypanosomal GMPR has a unique structure among species, and further structural analysis will be necessary to examine this possibility.

In the present study, we found the inhibitory effect of ribavirin on the proliferation of *T*. *brucei*. Ribavirin is widely used in the treatment of hepatitis C, and is believed to act as its nucleotide forms through the phosphorylation by intracellular kinase activities. We have previously reported that RMP is a potent inhibitor of TbIMPDH [11], in addition, we demonstrated here that RMP also inhibited TbGMPR more potently than its mammalian counterparts. Trypanosomes are known to have purine nucleoside transporters [33] and adenosine kinase [34,35], which participate in the RMP production from the extracellular ribavirin in the cells [36]. These findings suggest that ribavirin taken up to the parasite cells is converted to RMP and exerts its anti-trypaosomal activity through the inhibition of TbGMPR and/or TbIMPDH; however, another possibility still remains that ribavirin interferes the post-transcriptional modification via inhibiting the capping guanylation of mRNA [37]. Further investigation is required to ascertain whether these enzymes are essential to trypanosomes, and if proven so TbGMPR and/or TbIMPDH can be possible targets of ribavirin.

In conclusion, we clearly demonstrated in this study that GMPR of *T*. *brucei* apparently had characteristics distinct from those of its orthologs in the host animals and that the purine nucleotide analog RMP inhibited TbGMPR but not the host enzymes. Further studies to improve the potency and specificity of such inhibitors might lead to new therapeutics against African trypanosomiasis.

## Supporting Information

S1 Text Supporting Information Figure legends. (PDF)

S1 Fig Multiple alignment of GMPRs. Amino acid residues identical among the sequences are indicated on a black background. A shaded background represents the conserved amino acid residues in 5 or more sequences. TbGMPR possesses a tandem repeat of CBS domains (bars below the sequences), which are absent in human GMPR type1/2 (HsGMPR1/2), bovine GMPR type1/2 (BtGMPR1/2), and *E*. *coli* GMPR (EcGMPR). Circles and a triangle indicate GMP-binding residues and a catalytic Cys residue, respectively, reported previously for HsGMPR2 [1]. The sequence analysis was performed by the use of GENETYX software (Genetyx Co., Tokyo, Japan). The NCBI accession numbers are as follow: YP_001729062 for EcGMPR, NP_006868 for HsGMPR1, AAH03053 for HsGMPR2, NP_001069445 for BtGMPR1, and NP_001033208 for BtGMPR2.
(PDF)

S2 Fig Amino acid sequences of putative GMPRs of trypanosomatids. Amino acid residues identical among the sequences are indicated on a black background. A shaded background represents the conserved amino acid residues in 5 or more sequences. Note that the CBS domains (bars below the sequences) are conserved throughout the homologs of trypanosomatids represented. Peroxisomal targeting signal (PTS) sequences are indicated with a box. Gene IDs of the putative GMPRs in TriTrypDB are as follow: TcIL3000_5_1940 for *T*. *congolense*, TcCLB.508909.20 for *T*. *cruzi*, TevSTIB805.5.2400 for *T*. *evansi*, XP_003859941 (NCBI) for *L*. *donovani*, LinJ.17.0870 for *L*. *infantum*, and LmjF.17.0725 for L. major.
(PDF)

S1 Table Expect values in BLAST homology analysis between TbGMPR and GMPRs or IMPDHs of other organisms. (PDF)
