# Supplementary material for: Novel Characteristics of Trypanosoma brucei Guanosine 5'-monophosphate Reductase Distinct from Host Animals
Source: PLoS Negl Trop Dis. 2016 Jan 5;10(1):e0004339. doi: 10.1371/journal.pntd.0004339 (PMC4701174; doi:10.1371/journal.pntd.0004339)
Supplement: S1 Text — (PDF) [file pntd.0004339.s001.pdf]

S1 Text. Supporting information Figure legends

S1 Fig. Multiple alignment of GMPRs.

Amino acid residues identical among the sequences are indicated on a black background. A shaded background represents the conserved amino acid residues in 5 or more sequences. TbGMPR possesses a tandem repeat of CBS domains (bars below the sequences), which are absent in human GMPR type1/2 (HsGMPR1/2), bovine GMPR type1/2 (BtGMPR1/2), and *E. coli* GMPR (EcGMPR). Circles and a triangle indicate GMP-binding residues and a catalytic Cys residue, respectively, reported previously for HsGMPR2 [1]. The sequence analysis was performed by the use of GENETYX software (Genetyx Co., Tokyo, Japan). The NCBI accession numbers are as follow: YP_001729062 for EcGMPR, NP_006868 for HsGMPR1, AAH03053 for HsGMPR2, NP_001069445 for BtGMPR1, and NP_001033208 for BtGMPR2.

S2 Fig. Amino acid sequences of putative GMPRs of trypanosomatids.

Amino acid residues identical among the sequences are indicated on a black background. A shaded background represents the conserved amino acid residues in 5 or more sequences. Note that the CBS domains (bars below the sequences) are conserved throughout the homologs of

trypanosomatids represented. Peroxisomal targeting signal (PTS) sequences are indicated with a box. Gene IDs of the putative GMPRs in TriTrypDB are as follow: TcIL3000_5_1940 for *T. congolense*, TcCLB.508909.20 for *T. cruzi*, TevSTIB805.5.2400 for *T. evansi*, XP_003859941 (NCBI) for *L. donovani*, LinJ.17.0870 for *L. infantum*, and LmjF.17.0725 for L. major.
