# Supplementary material for: Novel Characteristics of Trypanosoma brucei Guanosine 5'-monophosphate Reductase Distinct from Host Animals
Source: PLoS Negl Trop Dis. 2016 Jan 5;10(1):e0004339. doi: 10.1371/journal.pntd.0004339 (PMC4701174; doi:10.1371/journal.pntd.0004339)
Supplement: S1 Fig — Amino acid residues identical among the sequences are indicated on a black background. A shaded background represents the conserved amino acid residues in 5 or more sequences. TbGMPR possesses a tandem repeat of CBS domains (bars below the sequences), which are absent in human GMPR type1/2 (HsGMPR1/2), bovine GMPR type1/2 (BtGMPR1/2), and E. coli GMPR (EcGMPR). Circles and a triangle indicate GMP-binding residues and a catalytic Cys residue, respectively, reported previously for HsGMPR2 [1]. The sequence analysis was performed by the use of GENETYX software (Genetyx Co., Tokyo, Japan). The NCBI accession numbers are as follow: YP_001729062 for EcGMPR, NP_006868 for HsGMPR1, AAH03053 for HsGMPR2, NP_001069445 for BtGMPR1, and NP_001033208 for BtGMPR2. (PDF) [file pntd.0004339.s002.pdf]

S1 Figure

```
TbGMPR    1:MSFNESASIPTGLTYDDVLIIPQHSRVTSRKEVNTT----TRLSRNVKLSIPIVASNMDT 56
EcGMPR    1:----MRIEEDLKLGFKDVLIRPKRSTLKSRSDVELERQFTFKHSGQSWSGVPIIAANMDT 56
BtGMPR1   1:---MPRIDADLKLDFKDVLLRPKRSSLKSRAEVDLERTFTFRNSKQTYSGIPIIVANMDT 57
BtGMPR2   1:---MPHIDNDVKLDFKDVLLRPKRSTLKSRSEVDLTRSFAFRNSKQMYTGIPIIAANMDT 57
HsGMPR1   1:---MPRIDADLKLDFKDVLLRPKRSSLKSRAEVDLERTFTFRNSKQTYSGIPIIVANMDT 57
HsGMPR2   1:---MPHIDNDVKLDFKDVLLRPKRSTLKSRSEVDLTRSFSFRNSKQTYSGVPIIAANMDT 57

TbGMPR   57:VCEQRMAVAMAREGGIGILHRFCSIEEQCAMLREVKRAQSFLIESPRIILPHETAREAWE 116
EcGMPR   57:VGTFSMASALASFDILTAVHKHYSVEEWQAFINNSS------------------------ 92
BtGMPR1  58:VGTFEMAVVMSQHSMFTAIHKHYTLDDWKLFAANH------------------------- 92
BtGMPR2  58:VGTFEMAKVLCKFSLFTAVHKHYSLEQWKEFASQN------------------------- 92
HsGMPR1  58:VGTFEMAAVMSQHSMFTAIHKHYSLDDWKLFATNH------------------------- 92
HsGMPR2  58:VGTFEMAKVLCKFSLFTAVHKHYSLVQWQEFAGRN------------------------- 92

TbGMPR  117:GLNWKGRVGGVGCLLVVNCKNERKLLGIITRHDLKLADESTTVESLMTPVDKMVVSTNTS 176
EcGMPR   92:------------------------------------------------------------ 92
BtGMPR1  92:------------------------------------------------------------ 92
BtGMPR2  92:------------------------------------------------------------ 92
HsGMPR1  92:------------------------------------------------------------ 92
HsGMPR2  92:------------------------------------------------------------ 92

TbGMPR  177:ISLEEVTHLMRKGRTANVPIVGQNGQLLYLVTLSDVVKLRKNKQASLDSRGRLLVGAAVG 236
EcGMPR   93:----------------------------------------------ADVLKHVMVSTGTS 106
BtGMPR1  93:----------------------------------------------PECLQHVAVSSGSG 106
BtGMPR2  93:----------------------------------------------PDCLEHLAASSGTG 106
HsGMPR1  93:----------------------------------------------PECLQNVAVSSGSG 106
HsGMPR2  93:----------------------------------------------PDCLEHLAASSGTG 106

TbGMPR  237:VKKDDMNRAIRLVEAGADVLVVDIAHGHSDLCINMVKRLKGDPRTASVDIIAGNIASAEA 296
EcGMPR  107:DADFEKTKQILDLNPALNFVCIDVANGYSEHFVQFVAKAR--EAWPTKTICAGNVVTGEM 164
BtGMPR1 107:KDDLEKMSNILEAVPQVKFICLDVANGYSEHFVEFVKLVR--SRFPEHTIMAGNVVTGEM 164
BtGMPR2 107:SSDFEQLEQILNAIPQVKYVCLDVANGYSEHFVEFVKDVR--KRFPEHTIMAGNVVTGEM 164
HsGMPR1 107:QNDLEKMTSILEAVPQVKFICLDVANGYSEHFVEFVKLVR--AKFPEHTIMAGNVVTGEM 164
HsGMPR2 107:SSDFEQLEQILEAIPQVKYICLDVANGYSEHFVEFVKDVR--KRFPQHTIMAGNVVTGEM 164

TbGMPR  297:AEALIDAGADGLKIGVGPGSICITRLVAGAGVPQLSAVLACTRVARRRGVPCIADGGLRT 356
EcGMPR  165:CEELILSGADIVKVGIGPGSVCTTRVKTGVGYPQLSAVIECADAAHGLGGMIVSDGGCTT 224
BtGMPR1 165:VEELILSGADIIKVGVGPGSVCTTRTKTGVGYPQLSAVIECADSAHGLKGHIISDGGCTC 224
BtGMPR2 165:VEELILSGADIIKVGIGPGSVCTTRKKTGVGYPQLSAVMECADAAHGLKGHIISDGGCSC 224
HsGMPR1 165:VEELILSGADIIKVGVGPGSVCTTRTKTGVGYPQLSAVIECADSAHGLKGHIISDGGCTC 224
HsGMPR2 165:VEELILSGADIIKVGIGPGSVCTTRKKTGVGYPQLGAVMECADAAHGLKGHIISDGGCNC 224

TbGMPR  357:SGDISKAIGAGADTVMLGNMLAGTDEAPGRVLVKDGQKVKIIRGMAGFGANLSKAERERT 416
EcGMPR  225:PGDVAKAFGGGADFVMLGGMLAGHEESGGRIVEENGEKFMLFYGMS-------SESAMKR 277
BtGMPR1 225:PGDVAKAFGAGADFVMLGGMFSGHTECAGEVIERNGQKLKLFYGMS-------SETAMKK 277
BtGMPR2 225:PGDVAKAFGAGADFVMLGGMLAGHSESGGELIERNGRKYKLFYGMS-------SEMAMKK 277
HsGMPR1 225:PGDVAKAFGAGADFVMLGGMFSGHTECAGEVFERNGRKLKLFYGMS-------SDTAMNK 277
HsGMPR2 225:PGDVAKAFGAGADFVMLGGMLAGHSESGGELIERDGKKYKLFYGMS-------SEMAMKK 277

TbGMPR  417:QDEDVFSSLVPEGVEGSVACKGPVGPIVRQLVGGLRSGMSYSGAKSIEEMQRRTRFVRMT 476
EcGMPR  278:HVGGVAEYRAAEGKTVKLPLRGPVENTARDILGGLRSACTYVGASRLKELTKRTTFIRVQ 337
BtGMPR1 278:HSGGVAEYRAPEGKTVEVPYKGDVENTILDILGGLRSTCTYVGAAKLKELSRRATFIRVT 337
BtGMPR2 278:YAGGVAEYRASEGKTVEVPFKGDVEHTIRDIIGGIRSTCTYVGAAKQKELSRRTTFIRVT 337
HsGMPR1 278:HAGGVAEYRASEGKTVEVPYKGDVENTILDILGGLRSTCTYVGAAKLKELSRRATFIRVT 337
HsGMPR2 278:YAGGVAEYRASEGKTVEVPFKGDVEHTIRDIIGGIRSTCTYVGAAKLKELSRRTTFIRVT 337

TbGMPR  477:GAGLRESGSHGVAKL--------------------------------------------- 491
EcGMPR  338:EQENRIFNNL-------------------------------------------------- 347
BtGMPR1 338:QQHNTVFS---------------------------------------------------- 345
BtGMPR2 338:QQVKPIFSDES------------------------------------------------- 348
HsGMPR1 338:QQHNTVFS---------------------------------------------------- 345
HsGMPR2 338:QQVNPIFSEAC------------------------------------------------- 348
```
