# Supplementary material for: Novel Characteristics of Trypanosoma brucei Guanosine 5'-monophosphate Reductase Distinct from Host Animals
Source: PLoS Negl Trop Dis. 2016 Jan 5;10(1):e0004339. doi: 10.1371/journal.pntd.0004339 (PMC4701174; doi:10.1371/journal.pntd.0004339)
Supplement: S2 Fig — Amino acid residues identical among the sequences are indicated on a black background. A shaded background represents the conserved amino acid residues in 5 or more sequences. Note that the CBS domains (bars below the sequences) are conserved throughout the homologs of trypanosomatids represented. Peroxisomal targeting signal (PTS) sequences are indicated with a box. Gene IDs of the putative GMPRs in TriTrypDB are as follow: TcIL3000_5_1940 for T. congolense, TcCLB.508909.20 for T. cruzi, TevSTIB805.5.2400 for T. evansi, XP_003859941 (NCBI) for L. donovani, LinJ.17.0870 for L. infantum, and LmjF.17.0725 for L. major. (PDF) [file pntd.0004339.s003.pdf]

S2 Figure

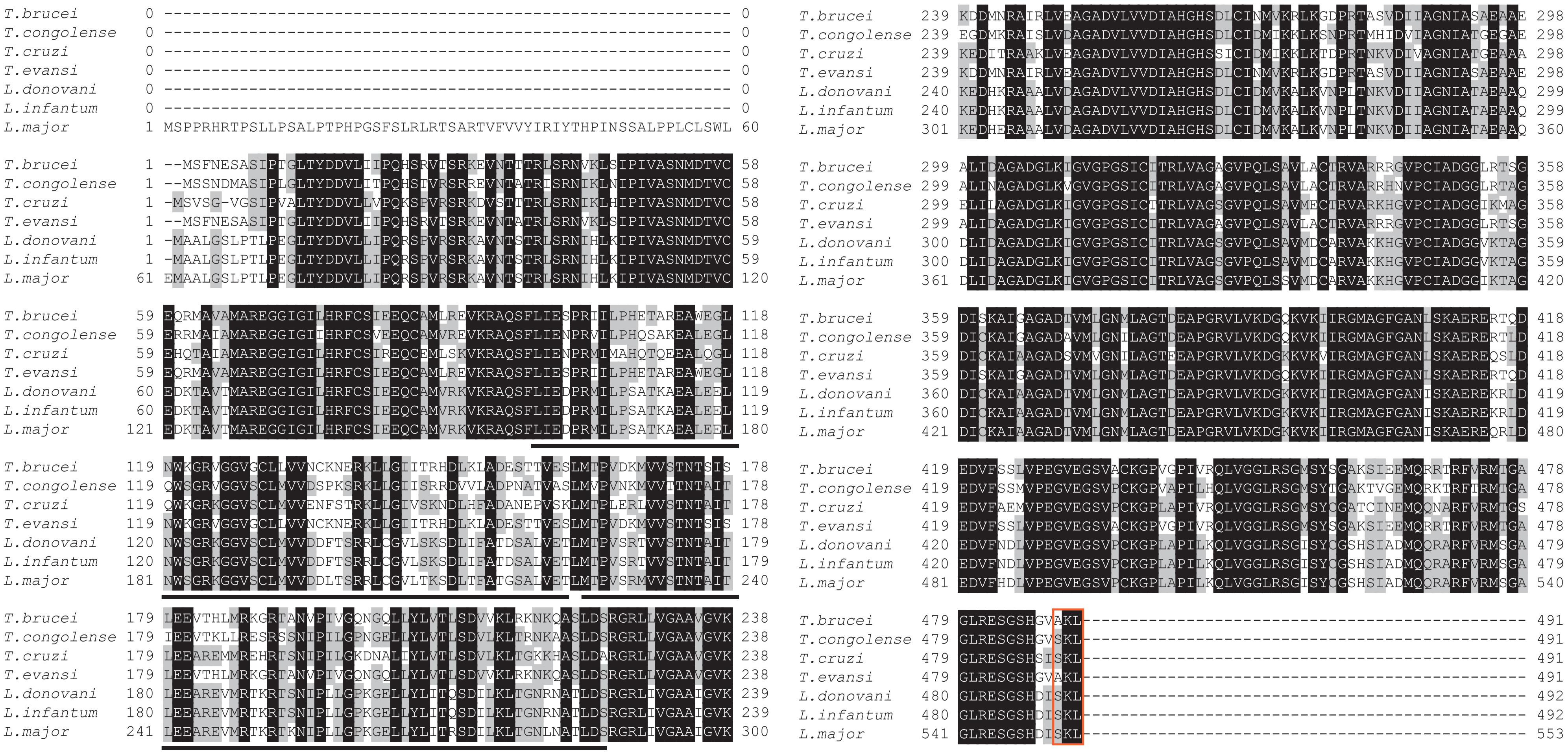
T.brucei 0 -- 0
T.congolense 0 -- 0
T.cruzi 0 -- 0
T.evansi 0 -- 0
L.donovani 0 -- 0
L.infantum 0 -- 0
L.major 1 MSPPRHRTPSLLPSALPTPHPGSFSLRLRTSARTVFVVYIRIYTHPINSSALPPLCLSWL 60
T.brucei 1 --MSFNESASIPTGLTYDDVLIIPQHSRVTSRKEVNTTTRLSRNVKLSIPIVASNMDTVC 58
T.congolense 1 --MSSNDMASIPLGLTYDDVLITPQHSTVRSRREVNTATRISRNIKLNIPIVASNMDTVC 58
T.cruzi 1 -MSVSG-VGSIPVALTYDDVLLVPQKSPVRSRKDVSTTTRLSRNIKLHIPIVASNMDTVC 58
T.evansi 1 --MSFNESASIPTGLTYDDVLIIPQHSRVTSRKEVNTATRLSRNVKLSIPIVASNMDTVC 58
L.donovani 1 -MAALGSLPTLPEGLTYDDVLLIPQRSPVRSRKAVNTSTRLSRNIHLKIPIVASNMDTVC 59
L.infantum 1 -MAALGSLPTLPEGLTYDDVLLIPQRSPVRSRKAVNTSTRLSRNIHLKIPIVASNMDTVC 59
L.major 61 EMAALGSLPTLPEGLTYDDVLLIPQRSPVRSRKAVNTSTRLSRNIHLKIPIVASNMDTVC 120
T.brucei 59 EQRMAVAMAREGGIGILHRFCSIEEQCAMLREVKRAQSFLIESPRIILPHETAREAWEGL 118
T.congolense 59 ERRMAIAMAREGGIGIIHRFCSVEEQCAMVREVKRAQSFLIENPRVILPHQSAKEALEGL 118
T.cruzi 59 EHQTAIAMAREGGIGILHRFCSIREQCEMLSKVKRAQSFLIENPRMIMAHQTQEEALQGL 118
T.evansi 59 EQRMAVAMAREGGIGILHRFCSIEEQCAMLREVKRAQSFLIESPRIILPHETAREAWEGL 118
L.donovani 60 EDKTAVTMAREGGIGILHRFCSIEEQCAMVRKVKRAQSFLIEDPRMILPSATKAEALEEL 119
L.infantum 60 EDKTAVTMAREGGIGILHRFCSIEEQCAMVRKVKRAQSFLIEDPRMILPSATKAEALEEL 119
L.major 121 EDKTAVTMAREGGIGILHRFCSIEEQCAMVRKVKRAQSFLIEDPRMILPSATKAEALEEL 180
T.brucei 119 NWKGRVGGVGCLLVVNCKNERKLLGIITRHDLKLADESTTVESLMTPVDKMVVSTNTSIS 178
T.congolense 119 QWSGRVGGVSCLMVVDSPKSRKLLGIISRRDVVLADPNATVASLMVPVNKMVVTTNTAIT 178
T.cruzi 119 QWKGRKGGVSCLMVVENFSTRKLLGIVSKNDLHFADANEPVSKLMTPLERLVVSTNTAIT 178
T.evansi 119 NWKGRVGGVGCLLVVNCKNERKLLGIITRHDLKLADESTTVESLMTPVDKMVVSTNTSIS 178
L.donovani 120 NWSGRKGGVSCLMVVDDFTSRRLCGVLSKSDLIFATDSALVETLMTPVSRTVVSTNTAIT 179
L.infantum 120 NWSGRKGGVSCLMVVDDFTSRRLCGVLSKSDLIFATDSALVETLMTPVSRTVVSTNTAIT 179
L.major 181 NWSGRKGGVSCLMVVDDLTSRRLCGVLTKSDLTFATGSALVETLMTPVSRMVVSTNTAIT 240
T.brucei 179 LEEVTHLMRKGRTANVPIVGQNGQLLYLVTLSDVVKLRKNKQASLDSRGRLLVGAAVGVK 238
T.congolense 179 IEEVTKLLRESRSSNIPILGPNGELLYLVTLSDVLKLTRNKAASLDSRGRLLVGAAVGVK 238
T.cruzi 179 LEEAREMMREHRTSNIPILGKDNALIYLVTLSDVLKLTGKKHASLDARGRLLVGAAVGVK 238
T.evansi 179 LEEVTHLMRKGRTANVPIVGQNGQLLYLVTLSDVVKLRKNKQASLDSRGRLLVGAAVGVK 238
L.donovani 180 LEEAREVMRTKRTSNIPLLGPKGELLYLITQSDIIKLTGNRNATLDSRGRLIVGAAIGVK 239
L.infantum 180 LEEAREVMRTKRTSNIPLLGPKGELLYLITQSDIIKLTGNRNATLDSRGRLIVGAAIGVK 239
L.major 241 LEEAREVMRTKRTKNIPLLGPKGELLYLITRSDIIKLTGNLNATLDSRGRLIVGAAIGVK 300
T.brucei 239 KDDMNRAIRLVEAGADVLVVDIAHGHSDLCINMVKRLKGDPRTASVDIIAGNIASAEAAE 298
T.congolense 239 EGDMKRAISLVDAGADVLVVDIAHGHSDLCIDMIKKLKSNPRTMHIDVIAGNIATGEGAE 298
T.cruzi 239 KEDITRAAKLVEAGADVLVVDIAHGHSSICIDMIKKLKTDPRTNKVDIVAGNIATGEAAA 298
T.evansi 239 KDDMNRAIRLVEAGADVLVVDIAHGHSDLCINMVKRLKGDPRTASVDIIAGNIASAEAAE 298
L.donovani 240 KEDHKRAAALVDAGADVLVVDIAHGHSDLCIDMVKALKVNPLTNKVDIIAGNIATAEAAQ 299
L.infantum 240 KEDHKRAAALVDAGADVLVVDIAHGHSDLCIDMVKALKVNPLTNKVDIIAGNIATAEAAQ 299
L.major 301 KEDHERAAALVDAGADVLVVDIAHGHSDLCIDMVKALKVNPLTNKVDIIAGNIATAEAAQ 360
T.brucei 299 ALIDAGADGLKIGVGPGSICITRLVAGAGVPQLSAVLACIRVARRRGVPCIADGGLRTSG 358
T.congolense 299 ALINAGADGLKVGVGPGSICITRLVAGAGVPQLSAVLACIRVARRHNVPCIADGGLRTAG 358
T.cruzi 299 ELILAGADGLKIGVGPGSICTTRLVAGSGVPQLSAVMECIRVARKHGVPCIADGGIKMAG 358
T.evansi 299 ALIDAGADGLKIGVGPGSICITRLVAGAGVPQLSAVLACIRVARRRGVPCIADGGLRTSG 358
L.donovani 300 DLIDAGADGLKIGVGPGSICITRLVAGSGVPQLSAVMDCARVAKKHGVPCIADGGVKTAG 359
L.infantum 300 DLIDAGADGLKIGVGPGSICITRLVAGSGVPQLSAVMDCARVAKKHGVPCIADGGVKTAG 359
L.major 361 DLIDAGADGLKIGVGPGSICITRLVAGSGVPQLSSVMDCARVAKKHGVPCIADGGIKTAG 420
T.brucei 359 DISKAIGAGADTVMLGNMLAGTDEAPGRVLVKDGQKVKIIRGMAGFGANISKAERERTQD 418
T.congolense 359 DICKAIGAGADAVMIGNILAGTDEAPGRVLVKDGQKVKIIRGMAGFGANISKAERERTLD 418
T.cruzi 359 DICKAIAAGADSVMVGNILAGTEEAPGRVLVKDGKKVKVIRGMAGFGANISKAEREQSLD 418
T.evansi 359 DISKAIGAGADTVMLGNMLAGTDEAPGRVLVKDGQKVKIIRGMAGFGANISKAERERTQD 418
L.donovani 360 DICKAIAAGADTVMLGNMLAGTDEAPGRVLVKDGKKVKIIRGMAGFGANISKAEREKRLD 419
L.infantum 360 DICKAIAAGADTVMLGNMLAGTDEAPGRVLVKDGKKVKIIRGMAGFGANISKAEREKRLD 419
L.major 421 DICKAIAAGADTVMLGNMLAGTDEAPGRVLVKDGKKVKIIRGMAGFGANISKAEREQRLD 480
T.brucei 419 EDVFSSLVPEGVEGSVACKGPVGPIVRQLVGGLRSGMSYSGAKSIEEMQRRTRFVRMIGA 478
T.congolense 419 EDVFSSMVPEGVEGSVPCKGPVAPILHQLVGGLRSGMSYIGAKTVGEMQRKTRFIRMIGA 478
T.cruzi 419 EDVFAEMVPEGVEGSVPCKGPLAPIVRQLVGGLRSGMSYCGATCINEMQQNARFVRMIGS 478
T.evansi 419 EDVFSSLVPEGVEGSVACKGPVGPIVRQLVGGLRSGMSYSGAKSIEEMQRRTRFVRMIGA 478
L.donovani 420 EDVFNDLVPEGVEGSVPCKGPLAPILKQLVGGLRSGISYCGSHSIADMQQRARFVRMSGA 479
L.infantum 420 EDVFNDLVPEGVEGSVPCKGPLAPILKQLVGGLRSGISYCGSHSIADMQQRARFVRMSGA 479
L.major 481 EDVFHDLVPEGVEGSVPCKGPLAPILKQLVGGLRSGISYCGSHSIADMQQRARFVRMSGA 540
T.brucei 479 GLRESGSHGVAKL-- 491
T.congolense 479 GLRESGSHGVSKL-- 491
T.cruzi 479 GLRESGSHSISKL-- 491
T.evansi 479 GLRESGSHGVAKL-- 491
L.donovani 480 GLRESGSHDISKL-- 492
L.infantum 480 GLRESGSHDISKL-- 492
L.major 541 GLRESGSHDISKL-- 553
