# Supplementary material for: Novel Characteristics of Trypanosoma brucei Guanosine 5'-monophosphate Reductase Distinct from Host Animals
Source: PLoS Negl Trop Dis. 2016 Jan 5;10(1):e0004339. doi: 10.1371/journal.pntd.0004339 (PMC4701174; doi:10.1371/journal.pntd.0004339)
Supplement: S1 Table — (PDF) [file pntd.0004339.s004.pdf]

S1 Tabale. Expect values in BLAST homology analysis between TbGMPR and GMPRs or IMPDHs of other organisms

GMPR	Organism (subtype)	IMPDH
4E-46	*Homo sapiens* (1)	6E-95
4E-45	*Homo sapiens* (2)	2E-98
5E-50	*Bos taurus* (1)	4E-97
2E-46	*Bos taurus* (2)	6E-98
4E-47	*E. coli*	2E-101

Numbers in parentheses indicate which subtype of GMPR or IMPDH was compared to TbGMPR. Note that the lower the expect value, the more "significant" the match is (http://blast.ncbi.nlm.nih.gov/Blast.cgi?CMD=Web&PAGE_TYPE=BlastDocs&DOC_TYPE=FAQ#expect).
